# Understanding the Mental Health of Students and Professors within Universities: a Cross-sectional, Multicultural Analysis across Three European Countries

**DOI:** 10.17533/udea.iee.v43n1e08

**Published:** 2025-04-28

**Authors:** Cristina García-Salido, Estella Ramírez-Baraldes, Felix Miedaner, Martina Hasseler, Andrea Hlubučková, Daniel Garcia-Gutierrez

**Affiliations:** 1 Nurse, Ph.D. Email: cgarcia@umanresa.cat https://orcid.org/0000-0002-9328-9477 Universitat de VIC Spain cgarcia@umanresa.cat; 3 Ph.D. Professor Faculty of Health Care and Health Care Sciences. Email: f.miedaner@ostfalia.de https://orcid.org/0000-0002-8931-2558 Spain f.miedaner@ostfalia.de; 4 Ph.D. Professor Faculty of Health Care and Health Care Sciences. Email: m.hasseler@ostfalia.de https://orcid.org/0000-0003-2337-9722 Germany m.hasseler@ostfalia.de; 5 University Professor, Psychologist, PhD. Email: hlubuckova@ivp.czu.cz https://orcid.org/0009-0009-8566-4925 Czech Republic (the) hlubuckova@ivp.czu.cz; 6 Nurse, Ph.D. Email: dgarcia04@umanresa.cat https://orcid.org/0000-0002-9909-7089 Universitat de VIC Spain dgarcia04@umanresa.cat; 7 Department of Nursing. Faculty of Health Sciences at Manresa. Central University of Catalonia (UVic-UCC), Manresa, Spain. https://orcid.org/0000-0002-9371-1754 Universitat de VIC Department of Nursing Faculty of Health Sciences at Manresa Central University of Catalonia (UVic-UCC) Manresa Spain; 8 Research Group on Simulation and Transformative Innovation (GRIST), Research and Innovation Institute in Life and Health Sciences of Central Catalonia (Iris-CC). Vic, Spain. Universitat de VIC Research Group on Simulation and Transformative Innovation (GRIST) Research and Innovation Institute in Life and Health Sciences of Central Catalonia (Iris-CC) Vic Spain; 9 Intensive Care Unit, Althaia, Xarxa Assistencial Universitària de Manresa, Fundació Privada, Manresa, Spain. Xarxa Assistencial Universitària de Manresa Intensive Care Unit Althaia Xarxa Assistencial Universitària de Manresa Manresa Spain; 10 Ostfalia University of Applied Sciences, Faculty of Health and Health Care Sciences, Wolfsburg, Germany. Ostfalia Hochschule für angewandte Wissenschaften Ostfalia University of Applied Sciences Faculty of Health and Health Care Sciences Wolfsburg Germany; 11 Czech University of Life Sciences Prague, Institute of Education and Communication, Prague, Czech Republic. Czech University of Life Sciences Prague Czech University of Life Sciences Prague Institute of Education and Communication Prague Czech Republic (the)

**Keywords:** mood, student health, university professor, COVID-19, pandemics., estado de ánimo, salud del estudiante, profesor universitario, COVID-19, pandemias, saúde do estudante, professor universitário, COVID-19, pandémies

## Abstract

**Objective.:**

To analyze the relationship among the professional role (student or professor), geographic location, and mental health in the university community after the COVID-19 pandemic.

**Methods.:**

Quantitative cross-sectional study conducted at universities in Spain, Germany, and the Czech Republic. Non-probability convenience sampling was used, obtaining a sample of 449 participants (372 students and 77 professors). Mental health was assessed using the Scale for Mood Evaluation (EVEA), measuring sadness-depression, anxiety, anger-hostility, and happiness.

**Results.:**

Significant differences were found between students and professors, with students reporting higher levels of sadness-depression (3.8 vs. 2.4; *p*<0.001), anxiety (4.6 vs. 2.9; *p*<0.001), and anger-hostility (3.4 vs. 2.5; *p*<0.01). Professors showed higher levels of happiness (6.7 vs. 5.4; *p*<0.001). In addition, differences among countries were observed: participants from Spain showed higher levels of sadness-depression and anxiety compared to Germany and the Czech Republic.

**Conclusion.:**

University students have greater emotional vulnerability than professors, highlighting the need for differentiated psychosocial support strategies in the academic setting.

## Resumen

**Objetivo.:**

Analizar la relación entre el rol profesional (estudiante o profesor), la ubicación geográfica y la salud mental en la comunidad universitaria tras la pandemia de COVID-19.

**Métodos.:**

Estudio transversal cuantitativo realizado en universidades de España, Alemania y República Checa. Se utilizó un muestreo no probabilístico de conveniencia, obteniendo una muestra de 449 participantes (372 estudiantes y 77 profesores). La salud mental se evaluó mediante la Escala de Evaluación del Estado de Ánimo (EVEA), midiendo tristeza-depresión, ansiedad, ira-hostilidad y felicidad.

**Resultados.:**

Se encontraron diferencias significativas entre estudiantes y profesores, con los estudiantes reportando niveles más altos de tristeza-depresión (3.8 vs. 2.4; *p*<0.001), ansiedad (4.6 vs. 2.9; *p*<0.001) e ira-hostilidad (3.4 vs. 2.5; *p*<0.01). Los profesores mostraron mayores niveles de felicidad (6.7 vs. 5,4; *p*<0.001). Además, se observaron diferencias entre países: los participantes de España presentaron mayores niveles de tristeza-depresión y ansiedad en comparación con Alemania y la República Checa.

**Conclusión.:**

Los estudiantes universitarios presentan mayor vulnerabilidad emocional que los profesores, lo que resalta la necesidad de estrategias diferenciadas de apoyo psicosocial en el ámbito académico.

## Resumo

**Objetivo.:**

Analisar a relação entre papel profissional (aluno ou professor), localização geográfica e saúde mental na comunidade universitária após a pandemia de COVID-19**.**

**Métodos.:**

Estudo transversal quantitativo realizado em universidades na Espanha, Alemanha e República Tcheca. Foi utilizada uma amostragem não probabilística por conveniência, obtendo-se uma amostra de 449 participantes (372 alunos e 77 professores). A saúde mental foi avaliada usando a Escala de Avaliação de Humor (EVEA), medindo tristeza-depressão, ansiedade, raiva-hostilidade e felicidade.

**Resultados.:**

Diferenças significativas foram encontradas entre alunos e professores, com os alunos relatando níveis mais altos de tristeza-depressão (3.8 vs. 2.4; *p*< 0.001), ansiedade (4.6 vs. 2.9; *p*< 0.001) e raiva-hostilidade (3.4 vs. 2.5; *p*< 0.01). Os professores apresentaram níveis mais elevados de felicidade (6.7 vs. 5.4; *p*< 0.001). Além disso, foram observadas diferenças entre os países: os participantes da Espanha apresentaram níveis mais altos de tristeza-depressão e ansiedade em comparação à Alemanha e à República Tcheca.

**Conclusão.:**

Estudantes universitários são mais vulneráveis ​​emocionalmente do que professores, o que evidencia a necessidade de estratégias diferenciadas de apoio psicossocial no âmbito acadêmico.

## Introduction

Detection of a new coronavirus (SARS-CoV-2) in Hubei province, China, in late 2019, and its rapid global spread prompted the World Health Organization (WHO) to declare COVID-19 a pandemic in March 2020. Although confinement and social distancing measures reduced transmission, these had negative repercussions on the population’s mental health, leading to increased symptoms of stress, anxiety, and depression.^1^ The academic environment was not immune to this crisis: the sudden transition from face-to-face teaching to the virtual or hybrid modality generated overload and emotional affectation in students and professors, given technological limitations and lack of planning.^2^ With the transition to the so-called "new normal" - a partial return to face-to-face teaching - fears arose about possible contagion, concern about learning backlogs and difficulties in adapting to restructured teaching methodologies. Numerous studies on student mental health have investigated risk factors, like the absence of support networks, living independently, and being the “first in the family” to attend university.^3^^,^^4^ For example, Horita *et al*.,^4^ reported that, although "high-risk" depression decreased in Japanese first-year students compared to previous cohorts, "academic distress" caused by virtual education increased. Similarly, in the United States, higher rates of anxiety, depression, and suicidal ideation were detected in those lacking social support or who assumed family responsibilities.^5^ Other works, such as those by Varela *et al*.,^6^ confirm that the pandemic impacted heterogeneously in different universities, evidencing the need to broaden the research perspective.

Despite increasing literature on mental health in the university population, the faculty staff remains a less explored group. Some indications point to the increased workload and emotional demand of professors, caused by the hasty adoption of technological tools and the need for greater accompaniment of students.^7^ Nevertheless, few comparative studies simultaneously assess the emotional well-being of professors and students, making it difficult to create tailored support strategies. This void becomes relevant when recalling that the professor’s mental well-being impacts upon the quality of teaching and, in turn, on students' performance.

On a more general level, university is described as a period of high susceptibility to mental disorders, particularly depression, anxiety, and substance use.^1^^-^^7^ The pandemic outbreak acted as a triggering or amplifying factor of such problems, underlining the urgency of institutional approaches to prevention and containment. The WHO’s Comprehensive Mental Health Action Plan 2013-2030 had warned about the importance of training undergraduate and graduate professionals to recognize and provide early care for mental health disorders.^8^ Similarly, research, such as that by Gestsdottir *et al*.,^9^ emphasizes the relevance of socioemotional support and the presence of protective factors, highlighting the need for contextualized interventions.

Within this context, focusing on nursing is especially important, given the strategic role of nurses in direct patient care, health education, and in promoting well-being within communities. Strong faculty and a mentally healthy student body are crucial for ensuring quality education, which - in turn - leads to enhanced clinical competencies and greater responsiveness to health emergencies. It is, therefore, necessary to understand how the pandemic affected both educators and learners to design effective psychoeducational interventions tailored to their needs. Are there differences in mental health among different European countries in the higher education setting two years after the onset of the COVID-19 pandemic, and does the emotional state of educators and students vary significantly within the same context?

To answer these questions, the aim of this study was to analyze the mental health of students and educators in three European countries, using the Scale for Mood Evaluation (EVEA), two years after the onset of the pandemic. This involves comparing sadness-depression, anxiety, anger-hostility, and happiness levels in both groups, examining the influence of sociodemographic factors, such as age, gender, or experience of isolation and assessing the implications of the results for nursing education and practice. Hopefully, this approach will contribute to outline strategies for the promotion and prevention of mental health in the university community, strengthening the quality of training of future health professionals and the resilience of health systems in the event of future crises.

## Methods

Study design and participants. A cross-sectional quantitative study was conducted in the faculties of Health and Life Sciences at universities in three European countries (the Czech Republic, Germany, and Spain). Non-probability convenience sampling was used, selecting participants from among students and professors who met the inclusion criteria and agreed to participate in the study. Thus, students could participate if they were enrolled in one of the study programs offered at the participating faculty during the 2022/2023 academic year. Faculty were included in the survey if they had been actively teaching in one of the programs offered since at least the 2019-2020 academic year (including the 2022/23 academic year). In addition, all participants had to be 18 years of age or older. Participants who did not complete the entire questionnaire and those who did not provide informed consent were excluded from the study. The response rate was 64.4%, ensuring a representative sample within the study context. The study was approved by the institutional ethics committees and was conducted in compliance with international human research regulations. Participants were informed of the study objectives, procedures, and their right to withdraw their participation at any time. Informed consent was obtained by means of an electronic form before answering the survey, guaranteeing their voluntary participation, the confidentiality of the data, and their treatment in accordance with the General Data Protection Regulation (GDPR) of the European Union. Due to differences in the accessibility of the participants and in the response rate per country, the distribution of the sample was not homogeneous. This aspect should be considered when interpreting the results and comparing among the countries evaluated.

Data collection. Data was collected via a self-administered electronic survey, conducted between November and December 2022. The questionnaire included: (i) sociodemographic information, such as professional role (student or professor), age, sex, country of origin, size of locality of residence, and experience of confinement during the COVID- 19 pandemic (March-June 2020); (ii) participants' mental health assessed by the Scale for Mood Evaluation (EVEA), developed by Sanz,^10^ designed to measure four clinically relevant emotional dimensions: sadness-depression, anxiety, anger-hostility, and happiness. The EVEA consists of 16 items, each composed of an 11-point Likert-type graphic scale (0 = not at all, 10 = very much), with statements beginning with the phrase "I feel" followed by an adjective reflecting the corresponding emotional state (*e.g*., "I feel sad", "I feel happy"). The adjectives used to assess sadness-depression and happiness come from the Spanish version of the Depression Adjective List,^11^ while the items for anxiety were taken from the State-Trait Anxiety Inventory.^12^ The anger-hostility subscale was elaborated from the translated version of the State-Trait Anger Scale.^13^ The EVEA has been validated in Spanish population and has been used in studies on cognition, emotional disorders, and mood assessment in clinical and educational settings.^14^^-^^16^ Its applicability in measuring the emotional impact of the pandemic has been confirmed in previous research.^17^

Regarding its psychometric properties, the EVEA has demonstrated adequate validity and reliability in prior studies. Herein, internal consistency, measured through Cronbach's alpha coefficient (α), was excellent for sadness-depression (α = 0.89) and anxiety (α = 0.90), and good for anger-hostility (α = 0.86) and happiness (α = 0.86).^17^ The specific values obtained in the Spanish sample, as well as in the subgroups of students and professors, are detailed in the results.

Statistical analysis. Analysis of variance (ANOVA) was used to test whether significant differences existed in each subscale between (i) the mental health of university students and professors and (ii) the mean mental health of participants in the different countries. A second step contrasted whether these differences remained significant after adjusting for selected sociodemographic variables (age, sex, size of residence and whether the participant was confined during the pandemic or not) using multiple linear regression models for each mental health subscale. Statistical analyses were performed with Stata V.17 (College Station, Texas, USA).

## Results

The survey was completed by 449 participants, distributed among 372 students (82.85%) and 77 professors (17.15%). The geographical distribution revealed that the majority of participants came from Spain (63.25%), followed by Germany (22.49%) and the Czech Republic (14.25%). In terms of age, 49.73% of the students were between 18 and 22 years old, reflecting a predominantly young sample. In contrast, most of the faculty were in higher age ranges, with a gradual increase in representation starting from age 33 onwards. With respect to gender, 59.91% of the sample identified as male, 39.64% as female, and 0.45% as intersex. Although the distribution within each group was relatively balanced, there was greater male representation among the students. Regarding the residential environment, 47.88% of the participants resided in cities with more than 60,000 inhabitants, while 23.83% lived in towns with less than 10,000 inhabitants. A relevant finding was the high prevalence of confinement experiences during the pandemic with 367 participants (81.73%) reporting being in isolation between March and June 2020.Table 1 presents a detailed breakdown of these sociodemographic characteristics, providing a more accurate picture of the composition of the sample.

**Table 1:** Descriptive statistics of the sample according to professional function

Variable	Students *n* (%)	Professors *n* (%)
Age in years		
18-22	185 (100)	0
23 - 27	94 (96.91)	3 (0.31)
28 - 32	22 (73.33)	8 (26.67)
33 - 37	18 (56.25)	14 (43.75)
38 - 42	18 (58.06)	13 (41.94)
43 - 47	18 (64.29)	10 (35.71)
48 - 51	12 (60)	8 (40)
52 - 57	5 (31.25)	11 (68.75)
58 - 61		4 (100)
62 - 65		4 (100)
> 65		2 (100)
Genre		
Male	219 (81.41)	50 (18.59)
Female	151 (84.83)	27 (15.17)
Intersex	2 (100)	
Country of origin		
Spain	225 (79.23)	59 (20.77)
Germany	90 (89.11)	11 (10.89)
Czech Republic	57 (89.06)	7 (10.94)
Number of inhabitants of the place of residence		
< 10.000	89 (83.18)	18 (16.82)
10.001 - 20.000	55 (88.71)	7 (11.29)
20.001 - 40.000	29 (80.56)	7 (19.44)
40.001 - 60.000	26 (89.66)	3 (10.34)
> 60.001	173 (80.47)	42 (19.53)
Have you been isolated because of Covid?		
No	70 (85.37)	12 (14.63)
Yes	302 (82.29)	65 (17.71)
Total	372 (82.85)	77 (17.15)

**Figure 1:**
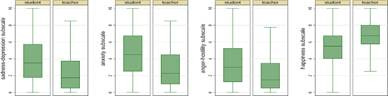
Mental health differences between students and professors

Based on the sample’s characterization, the differences in mental health between students and professors, as well as among the countries evaluated, were analyzed. Overall, it was noted that the mental health of the participants showed significant differences both between professors and students and among the countries analyzed. Analysis of variance (ANOVA) showed that mental health was significantly worse in students compared to professors in the four subscales assessed. In the sadness-depression dimension, students obtained a mean value of 3.8, while professors recorded 2.4 (*p*<0.001). Similarly, in the anxiety subscale, students presented a mean of 4.6, in contrast to 2.9 in professors (*p*<0.001). As for anger-hostility, students showed a mean value of 3.4 versus 2.5 in professors (*p*<0.01). Finally, in the happiness dimension, students obtained an average of 5.4, while professors reached 6.7. These results show that students experience significantly higher levels of sadness-depression, anxiety, and anger- hostility compared to professors. Moreover, professors reported higher levels of happiness, suggesting better emotional stability in this group. Figure 1 presents a visual summary of these differences, highlighting the gap in mental health between students and professors, as well as the variations among the countries evaluated. 

This Figure shows a clear difference in mental health levels between students and professors, with students having higher levels of sadness-depression, anxiety, and anger-hostility, and lower levels of happiness. However, when analyzing the differences according to country of origin, distinctive patterns are observed in all subscales, except for anger-hostility, where the differences were not significant. In particular, statistically significant differences were found in sadness-depression (p<0.01), anxiety (p<0.01), and happiness (p<0.001) among the countries evaluated

**Figure 2:**
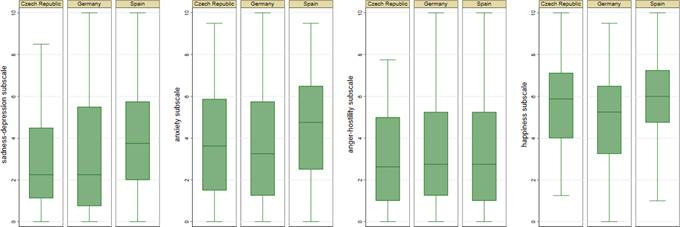
EVEA differences between students and professors by country

Figure 2 illustrates the differences in mental health between students and professors in each country, highlighting that Spain has the highest levels of sadness-depression and anxiety, while Germany and the Czech Republic report lower values in these dimensions. Nevertheless, happiness in the German participants was significantly lower compared to that of the Spanish participants.

After adjusting for sociodemographic variables, like age, gender, size of locality of residence, and experience of isolation during the pandemic, differences in mental health between students and professors remained statistically significant. Compared to professors, students had higher levels of sadness-depression (β = 1.157, *p*<0.01), anxiety (β = 1.373, *p*<0.01), and anger-hostility (β = 1.102, *p*<0.01), in addition to lower levels of happiness (β = -1.509, *p*<0.01). These findings suggest that students experience greater emotional vulnerability, possibly influenced by academic uncertainty, performance pressure, and less consolidation of coping strategies in crisis situations. Regarding differences among countries, taking Spain as the reference category, the results of the regression model indicated that participants from Germany and the Czech Republic reported significantly lower levels of sadness-depression and anxiety compared to their peers in Spain. Nonetheless, German participants also reported lower levels of happiness than those observed in Spain, suggesting the influence of sociocultural factors on the perception of emotional well-being.

**Table 2:** Results of multiple linear regression for each of the EVEA subscales

Variables	Sadness-depression β (EE)	Anxiety β (EE)	Anger-hostility β (EE)	Happiness β (EE)
Professional Function (Reference: professors)	1.157 (0.41)***	1.373 (0.419)***	1.102 (0.42)***)	-1.509 (0.319)***
Country (Reference: Spain)				
Germany	-0.883 (0.351)**	-0.966 (0.358)***	-0.113(0.359)	-0.731 (0.273)***
Czech Republic	-0.985 (0.4)**	-0.981 (0.409)**	-0.281(0.409)	0.007 (0.311)
Gender Reference: men)	0.12 (0.284)	0.091 (0.29)	0.015 (0.29)	0.1 (0.22)
Age	-0.111 (0.068)	-0.121 (0.07)*	0.046 (0.07)	-0.076 (0.053)
Size from city of residence. (Reference: <10 000 inhabitants)	0.08 (0.07)	0.134 (0.072)*	0.024 (0.072)	0.041 (0.055)
Isolation during the pandemic	0.179 (0.311)	0.171 (0.318)	0.266 (0.319)	-0.005 (0.242)
(Reference: Not isolated)				
Constant	2.69 (0.708)***	3.137 (0.723)***	1.932 (0.724)***	6.977 (0.55)***
Remarks	424	422	424	423
R-squared	0.092	0.111	0.022	0.095

* β = regression coefficient; SE = standard error. p < 0.05; ** p < 0.01; *** p < 0.001. Values without asterisks did not reach statistical significance. Percentages may not add up to exactly 100% due to incomplete responses.

## Discussion

The results of this study shed light on significant differences in emotional dimensions between students and professors, which may bear important implications to understand and address mental health and well-being in the educational setting. The following will discuss the main results and their relevance within the context of existing literature.

First, with respect to the sadness-depression dimension, it was observed that students reported significantly higher levels compared to professors. This finding is consistent with previous research that has noted the prevalence of depression in college students due to academic demands, life transitions, and social pressures. Early detection and intervention of sadness and depression among students may be essential to promote their emotional well-being and academic performance.^18^^,^^19^

Second, in relation to anxiety, the results indicate that students also experience higher levels of anxiety compared to professors. This discrepancy can be explained by the academic pressure to which students are exposed, such as exams, deadlines, and uncertainty about their future. Anxiety in the educational environment can affect negatively the quality of life and academic performance. Therefore, it is critical for educational institutions to implement anxiety management and support strategies for their students.^20^^,^^21^

Third, regarding the anger-hostility dimension, students reported higher levels than professors. This difference could be related to age and power dynamics in the classroom. The students may experience frustration and hostility due to lack of control over their educational environment and tensions among peers. Effective anger management and promotion of conflict resolution skills are important areas of emotional development within the student context.^22^ Finally, in terms of happiness, professors reported higher levels than students. This could be related to job satisfaction and accumulated teaching experience. Professors, having more experience in the educational system, may have developed effective strategies to cope with stress and maintain a work-life balance.^23^^,^^24^

The results herein have relevant implications for health sciences education and, particularly, for nursing education. The high prevalence of anxiety and depression symptoms in university students highlights the need to enhance psycho-emotional support programs within educational institutions. From a formative perspective, it is essential for nursing programs to incorporate strategies that foster emotional resilience and mental well-being in future health professionals. Likewise, given that professors reported higher levels of emotional well-being, their role as agents of support and containment in educational settings takes on special relevance. These findings suggest the importance of developing interventions focused on students and professors to optimize the quality of teaching and the training of health professionals with greater coping skills during crisis situations. In this sense, nursing, as a discipline, plays a fundamental role in the design and implementation of mental health promotion strategies within universities, which could contribute to improving the academic performance and emotional stability of future generations of health professionals.

Furthermore, our results reveal that both sadness-depression and anxiety were significantly related to the variables of professional role and geographic location in Spain. These results are consistent with previous research pointing to the influence of the role played by individuals on their emotional well-being. Specifically, participants who reported playing specific roles seem to experience higher levels of sadness-depression and anxiety. This could be related to the responsibilities and expectations associated with those roles. Additionally, the influence of geographic location, specifically Spain, on sadness-depression and anxiety is an intriguing finding. This could indicate the existence of cultural or contextual factors unique to Spain that affect the population’s emotional health.^25^^,^^26^

In contrast, the anger-hostility subscale only revealed a significant relationship with professional status (professors vs. students) with no significant differences among countries. This finding could suggest that the experience of anger and hostility is more linked to the perception of the role played by individuals than to geographical factors. It is important to further study how role expectations and role demands can trigger negative emotions such as anger.^27^

In terms of happiness, significant associations were found with occupational role and with the geographical locations of Spain and Czechia. Individuals in specific roles appeared to experience diverse levels of happiness, while participants from different regions, particularly Spain and Czechia, showed significantly divergent levels of happiness. These results suggest that individual and contextual factors significantly influence happiness; therefore, it is crucial to take into account a number of variables when examining emotional experiences and coping mechanisms, as well as to recognize sociocultural definitions of happiness. Overall, these results underscore the impact of personal and cultural factors on individuals' emotional well-being.^28^

From the differences between professors and students, suggesting that the mental health of professors is significantly better than that of students in all subscales assessed in this study, several reasons can be inferred. One possible explanation may be the different stressors faced by professors and students. While professors tend to have more stable working conditions and control over their work environment, students may face higher levels of uncertainty and pressure associated with academic demands and future employment.^7^ Another factor may be differences in experience and age group, which influence individual coping skills and emotions.^29^

Significant differences underscore the need to address and support the mental well-being of professors and students in educational settings. Finally, initial results suggest that the role played in the educational environment can have a significant impact on the mental health of individuals. Furthermore, these findings reinforce the idea that the academic environment not only influences student performance and training, but also impacts upon the mental health of those in the educational community. Grasping the influence of these factors is crucial for crafting university policies that prioritize emotional health. This study offers an empirical foundation for future research aimed at delving deeper into the determinants of well-being in educational settings and identifying the most effective strategies for its promotion.

This study has several limitations that should be considered when interpreting the results. First, its cross-sectional design precludes establishing causal relationships among professional role, geographic location, and mental health. Future longitudinal studies would make it possible to evaluate the evolution of emotional well-being in university communities. Second, the non-probabilistic convenience sampling limits the representativeness of the findings, restricting their generalizability to other university contexts. Expanding the sample in future research, by incorporating institutions from different countries and sociocultural contexts, would improve external validity. Also, the use of self-reports may have introduced social desirability biases and recall errors. Future studies could complement these data with psychophysiological measures or clinical assessments for greater objectivity. Another limitation was the lack of control over external factors, such as differences in academic load, psycho-emotional support systems, and access to mental health services, which could have influenced the results. Lastly, this study did not thoroughly investigate additional variables, such as teaching workload or student coping strategies, which are key aspects for a better understanding of emotional well-being in higher education.

Despite these limitations, the findings highlight the need for differentiated strategies to improve mental health in university settings and serve as a basis for future research with more robust methodological approaches.

The conclusion of this study is that significant differences exist in mental health between students and faculty members, with faculty consistently presenting lower levels of sadness-depression, anxiety, and anger-hostility and higher scores in happiness. These differences underscore the necessity of addressing university mental health with tailored strategies, taking into account the unique characteristics of each group and their effect on the quality of the educational process.

The results highlight that students show greater emotional vulnerability, underlining the importance of interventions aimed at strengthening their well-being. The high prevalence of depressive and anxious symptomatology in this group could be linked to academic and professional uncertainty, performance-related stress, and lack of consolidated coping strategies. In contrast, professors, although more emotionally stable, also face psychological challenges that may affect their performance and, therefore, the educational experience of students.

From an applied perspective, these findings have direct implications for health sciences education and nursing education. Promotion of emotionally healthy learning environments is critical to ensure the well-being of the student body and the quality of teaching. Thus, as a discipline, nursing should actively participate in developing strategies for the early identification of risk factors and the implementation of psychosocial intervention programs in university settings. Future studies should delve deeper into the factors that influence the relationship between the educational environment and mental health. Additionally, these should focus on developing preventive initiatives and support programs tailored to the needs of professors and students. This will not only enhance emotional well-being within the educational community but also boost the university system’s resilience in the face of future crises.

Future studies should explore the factors that influence the relationship between the educational environment and mental health. Furthermore, emphasis should be made on creating preventive initiatives and support programs specifically designed to meet the needs of professors and students. This will not only enhance emotional well-being within the educational community, but also bolster the university system’s resilience against future crises.
